# Textile weaving dataset for machine learning to predict rejection and production of a weaving factory

**DOI:** 10.1016/j.dib.2023.108995

**Published:** 2023-02-21

**Authors:** Toufique Ahmed, Shihab Uddin

**Affiliations:** a Department of Textile Engineering, National Institute of Textile Engineering and Research (NITER), Dhaka, Bangladesh; b Senior Manager (weaving), Evince Textiles Ltd., Gazipur, Bangladesh

**Keywords:** Weaving production data, Machine learning, Woven fabrics, Woven fabric rejection

## Abstract

Weaving is one of the most popular fabric manufacturing techniques. The weaving process consists of 3 major stages: warping, sizing, and weaving. The weaving factory henceforth involves a lot of data. But unfortunately, there is no attempt to utilize machine learning or data science in weaving production. Although a variety of scopes are there to implement statistical analysis, data science, and machine learning. The dataset was prepared by using the daily production report for 9 months. The final dataset contains 121,148 data with 18 parameters.  Whereas the raw data contains the same number of entries with 22 columns. The raw data needs substantial work to combine the daily production report, treat the missing values, rename columns, and feature engineering to derive EPI, PPI, warp, weft count values, etc. The complete dataset is stored at https://data.mendeley.com/datasets/nxb4shgs9h/1. It is further processed to get the rejection dataset which is stored at https://data.mendeley.com/datasets/6mwgj7tms3/2. The future implementation of the dataset is to predict the weaving waste, investigate the statistical relations among various parameters, production prediction, etc.

**Specifications Table**

**Table:** 

Subject	Textile Engineering

Specific subject area	Weaving (Woven fabric is a major fabric type. The woven fabrics are produced by weaving factories)
Type of data	Table
How the data were acquired	A weaving industry in Bangladesh named Evince Textiles Ltd. has been chosen considering diversified product manufacturing from various yarn counts. First, the daily production report from January to September (09 months) has been collected. Then the daily production reports were merged with the Pandas library and finally preprocess to get the final dataset. On average, each day's production report shows about 270 rows and 22 columns. Each day's production and rejection amount is a combination of 3 shifts (1 shift = 8 hours), Hence, the production report has been prepared by combining the 3 shifts. An officer collected the data from the batch card attached to the machine at the beginning of each shift's production. He also recorded the production quantity of each shift from the automatic LED display unit of the automatic loom. The daily production report is distributed to the managers and officers of the factory for an overall idea about the production status of different orders.
Data format	Raw analyzed Filtered
Description of data collection	The raw data (production record) was collected from Evince Textiles Ltd. from January 2013 to September 2013. The data were then merged, preprocessed, filtered, and feature-engineered to obtain the final dataset in CSV format.
Data source location	• Institution: Evince Textiles Ltd. • City/Town/Region: Gazipur, • Country: Bangladesh
Data accessibility	Dataset name: Full weaving dataset Repository name: Mendeley Data Direct URL: https://data.mendeley.com/datasets/nxb4shgs9h/1 Data identification number (DOI):10.17632/nxb4shgs9h.1 Dataset name: Weaving rejection dataset Repository name: Mendeley Data Direct URL to rejection data: https://data.mendeley.com/datasets/6mwgj7tms3/2 Data identification number (DOI):10.17632/6mwgj7tms3.2 Dataset name: Reference dataset and preprocessing code Repository name : Zenodo Direct URL: https://zenodo.org/record/7498062 Data Identification Number (DOI):10.5281/zenodo.7498062

**Value of the data**

• The textile industry has a lot of data, often the factory personnel look at the data and assume or predict something based on their experience. But if statistical tests were employed here then the prediction or assumption would be very accurate and effective.
• This dataset intends to build an algorithm to predict the weaving waste from some important clothing parameters such as yarn count, ends and picks per inch, and required quantity. Hence, the production manager may forcast the rejection amount of future woven fabric production.
• The presented dataset also helps to predict woven fabric production.
• The dataset can also find out the correlations among weaving production, yarn parameters, fabric rejections, etc.

## 1 Objective

Textile industries involve huge data due to the long interdependent processes. But there is very limited work on the implementation of machine learning in predicting or classifying fabric faults. Moreover, currently, the total production is estimated empirically or through machine speed. However, the rejection and production both depend on multiple factors such as yarn count, ends per inch (EPI), picks per inch (PPI), order length, etc. This dataset tends to facilitate the rejection or production prediction of a weaving industry.

## 2 Data Description

Woven fabric is one of the most commonly used fabric types. It is associated with a long process including warping, sizing, and weaving [1]. For weaving, typically modern air jet or rapier looms are used. An overview of the dataset entries is depicted in Table 1. The weaving management information department of the factory prepared daily production reports, which means each day has one production report. In this way for the month of January 31 production reports were available, for February it is 28, and so on. Each day's production report contains the date, order id, fabric construction, loom id (serial number of the used loom ), and details information about the yarn and fabric specifications. Each day on average 270 entries were recorded depending on the order quantity, loom stoppage due to mechanical and electrical problems, beam loading and unloading, and other problems. In this way, the total number of entries per month is also shown in Table 1.

**Table 1:** Total entries in the dataset with total daily production report.

Month	Total daily production report	Total entry in files
January	31	17,894
February	28	11,866
March	31	11,220
April	30	11,929
May	30 (1st May was International labor day)	13,073
June	30	16,095
July	31	13,185
August	31	10,451
September	30	15,435
Total data entry		121,148

But the production report (Table 2) is not useable for data analysis as it contains a lot of missing values (as the data entry officers intentionally keep the rows blanks to indicate the previous records). Besides, they used 3 rows as headers and many unnecessary data such as loom number, today's delivery, previous delivery, and total delivery which are irrelevant for machine learning and statistical analysis.

**Table:** 

Parameter	Description
ID	ID number of the fabric production order
construction	Warp count x weft count / EPI x PPI, a typical image of woven fabric is shown in Fig. 1 Warp count: yarn numbering (Ne) of the longitudinal yarn in woven fabrics. A higher yarn number means finer yarn. Weft count: yarn numbering of horizontal yarn in the woven fabrics. EPI: ends per inch (number of warp yarn per inch) PPI: picks per inch (number of weft yarn per inch) [2]
F.F Yds	Finished fabrics ordered in yards
F.A	Fabric Allowance
G.F Yds	Grey Fabrics in yards
Shrin._1%	Calculated shrinkage percent
B.L (M)	Beam length (length of yarn) in meter
L#	Loom number
Rec. B/L mtr	Received beam length in meter
Rec. B/L yds	Received beam length in yards
Todays pdn	Today's production
Previous pdn	Previous production
Total pdn in yds	Total production in yards, which is comprised of today's production and previous production
Shrin.%	Actual shrinkage percentage
Todays Del.	Today's delivery of grey fabrics
Pre.del	Previous delivery of grey fabrics
Total del	Total delivery of grey fabrics
Rej.& C.Pcs	Rejection and cut pieces
Total Prodn.	Total production
rejection%	Rejection in percentage
B/F B/C D/C	B/F: Beam finished, B/C: Beam change, D/C: Delivery Change
Remarks	Any other comments

**Table 2:** Daily production summary data: contains a lot of missing values, 3 rows as header, and unnecessary information .

Production summary													Date:- 01. April-13								Remarks
ID #	Construction	Order Quantity			Shrin._1%	B.L.(m)	L #	Rec.B/L mtr	Rec.B/L Yds	TODAYS Pdn	Previous pdn	TOTAL Prodn. Yds	Shrin.%	Grey (Inspected) Production					rejection%	B/F B/C D/C	
		F.F. Yds	F.A	G.F. Yds										Today's Del.	Pre. Del.	Total Del.	Rej.& C.Pcs	Total Prodn.			
13,064–5	40 × 40/110 × 70	15,136	7	16,275	10	16,536	144	3000	3280.8	0	2721	2721	17.06							B/F	
13,064–5(A)		1000	11.1	1124	12.5	1176	97	1145	1252.172	207	539	746	40.42								
							144	2500	2734	0	2279	2279	16.64							B/F	
							104	2900	3171.44	0	2579	2579	18.68							B/F	
							109	2500	2734	0	2271	2271	16.93							B/F	
							109	2885	3155.036	0	2594	2594	17.78							B/F	
								17,930	19,608.25		TOTAL	15,894	18.94	411	15,972	16,383	34	16,417	16		
13,068–2	40 × 40/120 × 70	1260	11.6	1425.3	12.7	1493	82	2600	2843.36	0	2332	2332	17.98							B/F	
13,073–3		2870	7.1	3089.3	11.2	3181	86	1600	1749.76	162	155	317	81.88

**Fig. 1:**
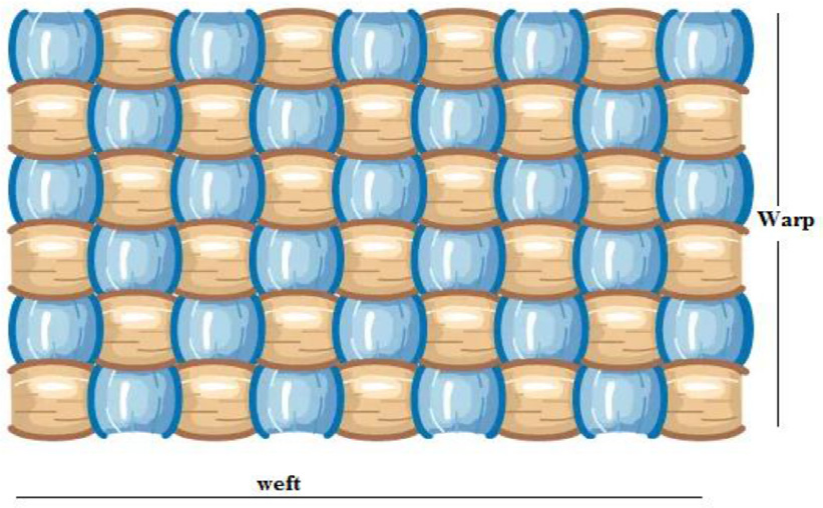
Warp and weft in Woven fabrics [3].

## 3 Experimental Design, Materials and Methods

The data was collected from the rapier loom (Leonardo, SOMET, and SMIT Brands, Italy) of Evince Textiles Ltd. These looms were fully automated and can keep records of all data related to production. The dataset used 272 days of daily production reports of 172 looms. The total entry of the production data (raw) was 121,148 with 22 columns.

The production report is not made for data analysis but to keep a record of the production. Hence, it contains a lot of unnecessary information, missing values, typing mistakes, and so on. As a result, we used different python libraries to preprocess it. Here, the Pandas data frame [4] was used as the main tool. The raw data and preparation code for this data has been uploaded to GitHub [5].

### 3.1 Preprocessing

First, the daily production reports (272 files for 272 days) were combined in a folder. Then all the files were merged into a single file using the Pandas library. The raw data [5] has some acronym columns that were renamed with meaningful ones. The primary dataset contains a lot of missing values, those were kept intentionally to imply the previous records. Hence, we filled the values with the previous ones. Then, some features engineering such as the required grey fabrics' length and beam length of the required grey fabrics were done. Again, the construction columns contain four very important pieces of information such as ends per inch (epi), picks per inch (ppi), warp count, and weft count. The information was split into 4 columns. Finally, the final dataset was achieved having 18 columns. From this dataset, two datasets were created one is the full weaving dataset [6] and another is for the rejection dataset [7]. The rejection dataset contains only the important columns and rows (22,010 rows and 14 columns) whereas the full dataset contains all information (121,148 rows and 18 columns). An example of the full dataset and rejection dataset is provided in Table 3 and Table 4, respectively. The full data set contains some Null values. These are for the special supplementary production where extra fabrics were needed to be produced but later due to the order fulfillment the looms remained idle, i,e there was no production but it was considered in the production dataset.

**Table 3:** Full weaving dataset (first and last 5 rows) : the na values here indicate that the values  are not available for these orders.

ID	Month	Construction	Req_Finish _Fabrics	Fabric_ Allowance	Rec_Beam_ length(yds)	assump_ crimp%	act_ crimp%	Previous_ pdn	Req_grey _fabric	Req_beam _length(yds)	Total_ pdn_m/c	Rej_ and _cut_ Piece	Total_ pdn _per _order	warp_ count	weft_count	epi	ppi
12,207–8	January	40+40/2/40/110 × 80	31,300	6	5752.336	12.5	12.26173	5047	33,297.87	34,797.65	5047	0	0	double	80	110	80
12,207–8	January	40+40/2/40/110 × 80	31,300	6	5883.568	12.5	64.12041	1952	33,297.87	34,797.65	2111	0	0	double	80	110	80
12,207–8	January	40+40/2/40/110 × 80	31,300	6	3094.888	12.5	24.13296	2207	33,297.87	34,797.65	2348	0	0	double	80	110	80
12,207–8	January	40+40/2/40/110 × 80	31,300	6	5894.504	12.5	14.73413	5026	33,297.87	34,797.65	5026	0	0	double	80	110	80
12,207–8	January	40+40/2/40/110 × 80	31,300	6	5850.76	12.5	21.46319	4391	33,297.87	34,797.65	4595	0	0	double	80	110	80
12,207–8	January	40+40/2/40/110 × 80	31,300	6	5905.44	12.5	22.69839	4340	33,297.87	34,797.65	4565	0	0	double	80	110	80
Last 5 rows																	
SF-13,277	September	40 × 40/100 × 90	83,449	7	109.36	10.2	na	na	89,730.10753	91,369.94215	na	0	0	40	40	100	90
SF-13,277	September	40 × 40/100 × 90	83,449	7	109.36	10.2	na	na	89,730.10753	91,369.94215	na	0	0	40	40	100	90
SF-13,277	September	40 × 40/100 × 90	83,449	7	109.36	10.2	na	na	89,730.10753	91,369.94215	na	0	0	40	40	100	90
SF-13,277	September	40 × 40/100 × 90	83,449	7	109.36	10.2	na	na	89,730.10753	91,369.94215	na	0	0	40	40	100	90
SF-13,277	September	40 × 40/100 × 90	83,449	7	109.36	10.2	na	na	89,730.10753	91,369.94215	na	0	0	40	40	100	90
SF-13,277	September	40 × 40/100 × 90	83,449	7	109.36	10.2	na	na	89,730.10753	91,369.94215	na	0	0	40	40	100	90

**Table 4:** Rejection dataset (first 5 rows).

Construction	Req_Finish _Fabrics	Fabric_ Allowance	Rec_Beam _length(yds)	Shrink_allow	Previous _pdn	Req_grey_fabric	Req_beam_length(yds)	Total_Pdn(yds)	Rejection	warp_count	weft_count	epi	ppi
40+40/2/40/ 110 × 80	31,300	6	5752.3	12.5	TOTAL	33,297.87	34,797.65	5047	285	Double _40	80	110	80
40 × 40/110 × 90	31,300	6	5883.5	12.5	TOTAL	33,297.87	34,797.65	2111	39	40	80	110	90
40 × 40/110 × 80	31,300	6	3094.8	12.5	TOTAL	33,297.87	34,797.65	2348	0	40	80	110	80
40 × 40/130 × 80	31,300	6	5894.5	12.5	TOTAL	33,297.87	34,797.65	5026	58	40	80	110	80
50 × 50/140 × 70	31,300	6	5850.7	12.5	TOTAL	33,297.87	34,797.65	4595	1043	50	80	110	70

Describing the parameters:

**Table:** 

Parameter	Description
Req_Finish_Fabrics	Required finished fabrics. It consists total amount of required fabrics in length and the allowance for shrinkage and wastage.
Fabric_Allowance	Fabric allowance: Due to different cases such as wastage, shrinkage, and extra fabric due to safety margins.
Rec_Beam_length(yds)	Received beam length (yards): total length of yarn supplied from the production planning department
assump crimp%	Assumed crimp percentage
act_crimp	actual crimp
Previous_pdn	Production of the previous shift
Req_grey_fabric	Calculated grey fabrics. It should be more than the order. Required grey fabrics = $\frac{\text{finished}\;\text{fabrics}\;\times \;100}{\left(100-\text{fabric}\;\text{allowance}\right)}$
Req_beam_length(yds)	Calculated warp yarn for the total required fabrics including shrinkage, allowance, etc. Required beam length = $\frac{\text{required}\;\text{grey}\;\text{fabrics}\;\times \;100}{\left(100-\text{shrink}\_\text{allow}\right)}$
Total_Pdn(yds)	Sum of the production of different shifts
Rej_and_cut_Piece	Rejection and cut piece. The amount of rejected fabrics due to fabrics faults such as damage, floating of yarn, loose picks, pattern mismatch, etc.
Total_pdn_per_order	Total production per order
warp_count	Warp count
Weft_count	Weft count
epi	Ends per inch
ppi	Picks per inch

## Ethics Statement

The data of this article involve neither animal nor human participants. Besides, according to the company's data distribution policy data can be shared for research and non-commercial purposes. Hence our dataset complies with the data distribution policy of the company.

## CRediT authorship contribution statement

**Toufique Ahmed:** Conceptualization, Methodology, Writing – original draft, Software. **Shihab Uddin:** Resources, Data curation.

## Declaration of Competing Interest

The authors declare that they have no known competing financial interests or personal relationships that could have appeared to influence the work reported in this paper.

## Data Availability

Full weaving dataset (Original data) (Mendeley Data). weaving rejection dataset (Original data) (Mendeley Data). weaving dataset preparation code (Reference data) (Zenodo).
